# Causal inference of sex hormone-binding globulin on venous thromboembolism: evidence from Mendelian randomisation

**DOI:** 10.1186/s12959-023-00553-9

**Published:** 2023-10-25

**Authors:** Shuping Wang, Yongxiang Wang, Ming Bai, Yu Peng, Dan Zhou, Peng Lei, Binpeng Zhou, Piyi Zhang, Zheng Zhang

**Affiliations:** 1 The First Clinical Medical School, Lanzhou University, Lanzhou, Gansu China; 2 Heart Center, The First Hospital of Lanzhou University, Lanzhou, Gansu China; 3 Gansu Provincial Clinical Research Center for Cardiovascular Diseases, The First Hospital of Lanzhou University, Lanzhou, Gansu China; 4 Gansu Key Laboratory of Cardiovascular Diseases, The First Hospital of Lanzhou University, Lanzhou, Gansu China; 5 Department of Emergency, The First Hospital of Lanzhou University, Lanzhou, Gansu China

**Keywords:** Sex hormone-binding globulin, Testosterone, Venous thromboembolism, Deep vein thrombosis, Pulmonary embolism, Mendelian randomisation, Instrumental variables

## Abstract

**Background:**

Previous cohort studies have shown that exogenous sex hormone use, such as testosterone replacement therapy and oestrogen-containing contraceptives, can increase the risk of venous thromboembolism (VTE). However, the relationship between endogenous sex hormone levels and VTE remains unclear. The goal of the present study was to explore the causal roles of endogenous sex hormones, including hormone-binding globulin (SHBG), bioactive testosterone (BT), and total testosterone (TT), in VTE and its two subgroups, deep vein thrombosis (DVT) and pulmonary embolism (PE).

**Methods:**

We used a genome-wide association study of sex hormones as exposure data and Finnish VTE data as the outcome. Inverse variance weighting, MR-Egger, and weighted median were used for two-sample Mendelian randomisation (MR). Sensitivity analyses included MR-Egger, MR-PRESSO, Cochrane Q test, MR Steiger, leave-one-out analysis, and funnel plot, combined with multivariate MR and replicated MR analyses using larger VTE data from the global biobank meta-analysis initiative. Linkage disequilibrium score regression (LDSC) was used to determine genetic associations and estimate sample overlap.

**Results:**

Our findings genetically predicted that an increase in serum SHBG levels by one standard deviation (SD) caused 25% higher odds for VTE (OR: 1.25, 95% CI: 1.01−1.55) and 58% higher odds for PE (OR: 1.58, 95% CI: 1.20−2.08). LDSC supported the genetic correlation between these two traits and replicated analyses confirm SHBG’s genetic effect on VTE in both sexes (OR: 1.46, 95% CI: 1.20−1.78) and in females (OR: 1.49, 95% CI: 1.17−1.91). In addition, an increase in serum TT levels by one SD caused 32% higher odds for VTE (OR: 1.32, 95% CI: 1.08−1.62) and 31% higher odds for DVT (OR: 1.31, 95% CI: 1.01−1.69); however, LDSC and replicated analyses did not find a genetic correlation between TT and VTE or its subtypes. No significant correlation was observed between BT and all three outcome traits.

**Conclusion:**

Our study provides evidence that elevated serum SHBG levels, as predicted by genetics, increase VTE risk. However, the causal effect of testosterone levels on VTE requires further investigation.

**Supplementary Information:**

The online version contains supplementary material available at 10.1186/s12959-023-00553-9.

## Background

Venous thromboembolism (VTE), which includes interrelated diseases such as deep vein thrombosis (DVT) and pulmonary embolism (PE), is among the top five most common vascular diseases in many regions [1]. In Western populations, the lifetime incidence of VTE is approximately one in 12 individuals. The survival rate after VTE diagnosis is much lower than expected; approximately 20% of individuals die within 1 year of VTE diagnosis, from VTE or other cardiovascular diseases, neoplastic conditions, or respiratory system disorders; and complications tend to manifest frequently in survivors [2–4]. The pathogenesis of VTE is complex and involves interactions among genetic, environmental, and lifestyle factors. Identifying underlying causal factors is important for developing effective preventive and therapeutic strategies.

Exogenous sex hormones such as hormone replacement therapy and oral oestrogen-containing contraceptives increase the risk of VTE in women [3, 5]. Additionally, some studies have shown that testosterone therapy may increase the risk of VTE in men [6, 7]. Regarding endogenous sex hormones, substantial evidence showed that endogenous oestrogen can reduce thrombosis by regulating coagulation and inflammation and inhibiting the vascular injury response [8, 9]. However, limited studies are available on the impact of endogenous testosterone and sex hormone-binding globulin (SHBG) on the risk of VTE. A prospective study incorporating 3,051 postmenopausal women and 3,925 middle-aged to older men without hormone replacement therapy (HRT) suggested that endogenous testosterone was not associated with VTE risk in a median follow-up of 17.6 years [10]. An analogous prospective study incorporated 4658 women and 4673 men and found that endogenous testosterone was not associated with VTE and its two subtypes during a follow-up period of 21 years [11]. However, the effects of SHBG on VTE risk remain unclear. Since its identification by Mercier et al. in 1966 [12], SHBG has been regarded as a glycoprotein synthesised in the liver which exhibits a strong binding affinity to circulating sex hormones, including 5α-dihydrotestosterone, testosterone, and 17β-oestradiol protein, and plays a crucial role in regulating their bioavailability at the target location [13–15]. However, recent studies have yielded new perspectives on the role of SHBG, suggesting that it can serve as both a biomarker and a potential drug candidate for multiple diseases, independent of sex hormones [16–18]. Odlind et al. first proposed SHBG as a standalone prognosticator of VTE risk following the administration of hormonal contraceptives [19], and subsequent studies supported this view [20, 21]. A case-control study conducted by Luuk et al. suggested that the risk of VTE shows a dose-dependent response to serum SHBG levels in women aged ≤ 45 years (369 cases and 296 controls) without exposure to exogenous hormones or pregnancy [22]. In addition, Raps et al. suggested that SHBG can be a risk marker of VTE for contraceptive preparations based on the positive correlation between SHBG and the normalized activate protein C (APC) sensitivity ratio and VTE risk in contraceptive users [20]. However, some studies suggested that SHBG has no significant effect on VTE. The aforementioned prospective study found no significant correlation between endogenous SHBG levels and VTE [10]. Frank et al. suggested that SHBG levels are not a useful risk marker for VTE in women using oral contraceptives [23]. Therefore, it remains unclear whether endogenous testosterone and SHBG levels are associated with VTE risk. Although some observational studies have small sample sizes, their results are contradictory and may be confounded by multiple confounding factors as well as reverse causalities. Moreover, the causal relationships between SHBG, endogenous testosterone, and VTE remain unclear. Hence, there is a need for a rigorous investigation of the causal effects of SHBG and testosterone levels on VTE risk.

Mendelian randomisation (MR) is a potent statistical method that establishes causal links between exposures and outcomes using single nucleotide polymorphisms (SNPs) as instrumental variables (IVs) [24]. In contrast to observational studies, MR can provide more robust evidence of causality by circumventing the intrinsic limitations of these studies, such as the risk of confounding, reverse causation, and measurement errors, by leveraging randomly assigned genetic variants during gametogenesis [25]. Although randomised controlled trials provide the best clinical evidence compared with MR research, they have limitations such as large investment in manpower and material resources, long follow-up time, and difficulty in implementation [25]. Therefore, the objective of this study was to further explore the potential causal effects of SHBG and testosterone on VTE, including its two subtypes, PE and DVT, using two-sample MR within the largest genetic population samples of SHBG and testosterone.

## Methods

### Study design

**Fig. 1:**
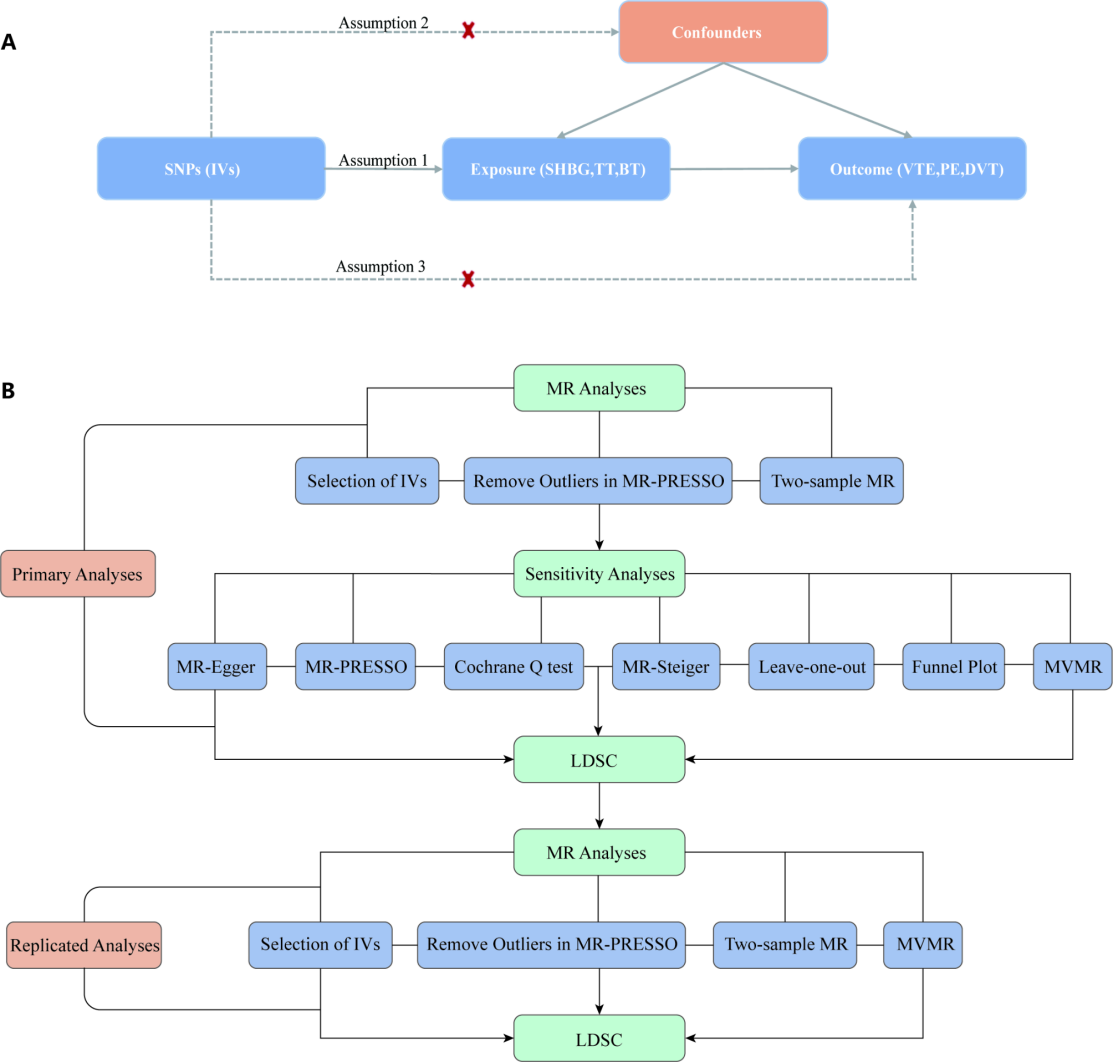
Diagram of the two-sample MR study **(A)** and the study design **(B)**. MR: Mendelian randomisation; IVs: Instrumental variables; SNPs: Single nucleotide polymorphisms; SHBG: Sex hormone-binding globulin; TT: Total testosterone; BT: Bioactive testosterone; VTE: Venous thromboembolism; PE: Pulmonary embolism; DVT: Deep vein thrombosis; LDSC: Linkage disequilibrium score regression; MVMR: Multivariable Mendelian randomisation

We conducted a two-sample MR study to assess whether there is a causal effect of genetically predicted SHBG, total testosterone (TT), and bioactive testosterone (BT) on VTE and its subtypes PE and DVT. MR analysis utilizes genetic variation as a source of natural randomisation, and SNPs, which represent worldwide human genetic diversity, were chosen as IVs that are not subject to environmental or behavioural selection bias. There were three fundamental hypotheses of the MR analysis: (1) IVs are highly correlated with the exposure variable; (2) IVs remain unaffected by any confounding factors; and (3) IVs only impact the outcome variable through the exposure variable. This includes relevance, independence, and exclusion restrictions [26]. A diagram of the two-sample MR study is shown in Fig. 1A. Second, MR sensitivity analyses were performed using MR-Egger, MR-PRESSO, Cochrane Q test, MR-Steiger, leave-one-out, funnel plot, and Multivariable Mendelian randomisation MR (MVMR). Then, replicated MR analyses using larger VTE data from the Global Biobank Meta-analysis Initiative (GBMI) were performed to verify the results of the primary MR analyses. Finally, linkage disequilibrium score regression (LDSC) was used to determine genetic associations and estimate sample overlap. A diagram of the study design is shown in Fig. 1B.

### Exposure data source

The exposure data for SHBG, TT, and BT were procured from publicly available genome-wide association studies (GWAS) provided by Ruth et al. [16] using data from UK Biobank, a national resource that has been described extensively elsewhere (https://www.ukbiobank.ac.uk) [27]. Individuals aged 40 to 69 years (men and women), who were enrolled in the National Health Service and residing within a maximum distance of 25 miles from any of the 22 designated study assessment centres, were eligible for participation between 2006 and 2010 [16]. Serum SHBG and TT levels were quantified in nmol/L using a two-step sandwich immunoassay analysis and one-step competitive analysis, respectively, in 425,097 participants of European ancestry. BT (N = 382,988) was calculated as nmol/L from albumin and TT levels [16]. Additionally, given that previous studies have shown relationships between body mass index (BMI) and SHBG as well as between BMI and VTE, we used BMI-adjusted SHBG data provided by the authors to mitigate the influence of BMI as a potential confounder on the study’s outcomes.

### Outcome data source

The outcome data on VTE were sourced from the R8 version of the FinnGen GWAS database (https://r8.finngen.fi, date of retrieval: 2023-03-25), which contains electronic health record data on over five million Finnish individuals, including hospital discharge diagnoses and prescribed medication information. The phenotypic codes for VTE, DVT, and PE were ‘I9_VTE’ (17,048 cases, 325,451 controls), ‘I9_PHLETHROMBDVTLOW’ (8.077 cases, 295,014 controls), and ‘I9_PULMEMB’ (8,170 cases, 333,487 controls), respectively. The FinnGen GWAS database incorporated a VTE population aged 10 to 100 years. The median ages at the first event were 60.20, 57.80, and 62.96 years for both sexes, women, and men, respectively, in VTE cases; 58.86, 56.99, and 60.84 years, respectively, in DVT cases; and 65.50, 64.28, and 66.76 years, respectively, in PE cases (https://risteys.finregistry.fi/). The diagnoses of these VTE events were defined using nationwide registries of hospital discharge and cause of death data, using the International Classification of Diseases (ICD) revisions 8, 9, and 10 (https://risteys.finregistry.fi/) [28]. Replication was performed using another larger VTE dataset with 915,868 participants of European ancestry provided by the Global Biobank Meta-analysis Initiative (GBMI, https://www.globalbiobankmeta.org/, date of retrieval: 2023-04-15). To initially explore the causal relationship between SHBG, testosterone, and VTE in specific sexes, we further used the female VTE dataset from the GBMI as the outcome for verification, which contained 454,926 female participants, mostly of European ancestry (92.4%) [29]. The disease endpoints in the GBMI were defined following the ICD-9 or ICD-10 codes. The average age of all participants in the GBMI was 39.91–69.86 years, and the standard deviation of age was 7.9–25 years; however, the GBMI did not provide age span information for the VTE population specifically [29].

### Selection of instrumental variables

To select SNPs that could serve as valid genetically predicted IVs for SHBG and testosterone, we initially established a rigorous significance threshold of *P* < 5 × 10^− 8^. Second, to avoid linkage disequilibrium, we grouped SNPs (r^2^ ≤ 0.001 and kb = 10,000). Subsequently, SNPs with an F-statistic < 10 were excluded. The F-statistic for each SNP was computed using the formula F = (N-2) × R^2^ / (1-R^2^), where N represents the sample size, and R^2^ represents the proportion of variance in the exposure variable explained by each SNP. For palindromic SNPs, researchers have attempted to deduce positive-strand alleles by utilizing allele frequencies, removing SNPs with intermediate allele frequencies because their orientation concerning exposure and outcome in GWASs cannot be determined with certainty. The resulting SNPs were used as IVs in the MR analysis and the overall R^2^ of the IVs for each trait pair was calculated to assess the efficacy of the IVs.

### Mendelian randomisation analysis

The analyses were performed using R software version 4.2.3, using the ‘Two-Sample MR’ and ‘MR-PRESSO’ packages [30]. Multiple MR methods have been used to examine causal relationships, including inverse variance weighting (IVW) [31], MR-Egger [32], weighted median [33], weighted mode, simple mode, and MR-pleiotropy residual sum and outlier (MR-PRESSO) [34]. Each method assumes different criteria for instrumental variable (IV) validity; however, the IVW method is widely regarded as the most robust approach. Essentially, IVW is a meta-analysis technique that integrates causal estimates from each IV through weighted aggregation to obtain the final estimate of the causal effect. Therefore, in this study, IVW was used as the principal methodology to ascertain the causal associations between exposure and outcome. The remaining methods were utilized as ancillary approaches to complement the main analysis or offer supplementary insights. If MR-PRESSO identified any outlier SNPs, they were initially excluded, followed by a reassessment of the remaining IVs. Scatter plots were constructed to show the causal estimates of the five MR methods. Single SNP analyses were performed and forest and funnel plots of the IVW and MR-Egger methods were used to describe the results. Odds ratios (ORs) and 95% confidence intervals (CI) were used to quantify their influence on VTE risk. A significance level of *P* < 0.05 was deemed indicative of a potential association. The Benjamini-Hochberg (BH) procedure was employed to control the false discovery rate for multiple comparisons by adjusting the *P*-values. To rule out reverse causality, we conduct an MR-Steiger directionality test.

### Sensitivity analysis

For sensitivity analysis, we first employed two other MR methods, MR-Egger and median MR, which are known for their increased robustness against pleiotropy. Directionally concordant outcomes with IVW strengthened the validity of our causal inferences. We also used the MR-PRESSO test, which identifies and corrects for pleiotropy by detecting and removing outlier SNPs that violate the assumption of no horizontal pleiotropy [34]. Finally, we assessed pleiotropy by conducting a leave-one-out analysis wherein we systematically excluded each SNP and assessed its impact on the causal estimate. Cochrane’s Q-statistic, derived from the IVW method, was used to assess the heterogeneity among the estimates obtained from individual SNPs. Moreover, LDSCs have been used to assess genome-wide genetic associations between exposure and outcomes. Intercepts from the LDSC also indicated a potential sample overlap between the two GWAS. Finally, the two-sample MR results were validated by using an independent outcome dataset with a larger sample size. MVMR was performed to rule out bias due to the correlation between the sex hormone levels.

## Results

### Selection of instrumental variables

IVs for SHBG, TT, and BT were selected based on established quality control criteria after clumping and excluding SNPs with an F-statistic of less than 10, suggesting that the presence of a weak IV bias is unlikely to exert a significant effect. After harmonization of the alleles and effects between the exposure and outcome in primary analyses, there were 148,148,147 SNPs used for causal analyses of SHBG with VTE, PE, and DVT (R^2^ = 0.062); 138,140,140 SNPs for TT with VTE, PE, and DVT (R^2^ = 0.027); and 86,89,91 SNPs for BT with VTE, PE, and DVT (R^2^ = 0.019). In the replicated analyses, there were 147 SNPs used for causal analysis of SHBG with VTE (R^2^ = 0.060), 149 SNPs for TT with VTE (R^2^ = 0.031), 93 SNPs for BT with VTE (R^2^ = 0.019), 88 SNPs for SHBG in women (SHBGw) with VTE in women (VTEw) (R^2^ = 0.105), 193 SNPs for TT in women (TTw) with VTEw (R^2^ = 0.075), and 127 SNPs for BT in women (BTw) with VTEw (R^2^ = 0.052). The detailed characteristics of these IVs are shown in Additional File 1 (Supplementary Table S1).

### Causal estimates of genetically predicted SHBG, TT and BT with VTE

**Fig. 2:**
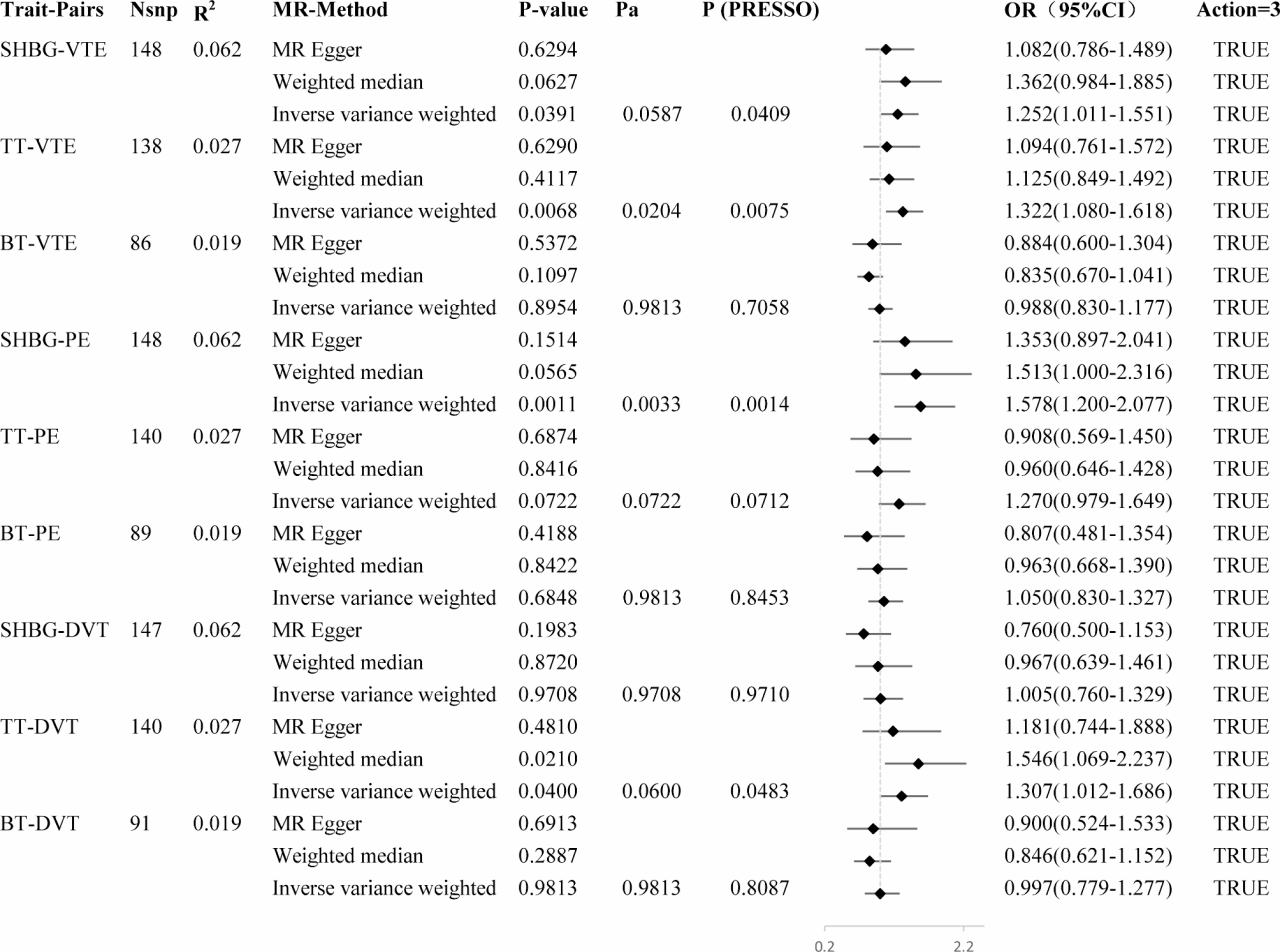
Causal relationships between sex hormones and VTE in primary two-sample MR analyses. The effect of a one-standard deviation (SD) increase in sex hormone levels on the odds ratio (OR) of VTE is represented by the OR and its corresponding 95% confidence interval (CI). MR: Mendelian randomisation; Nsnp: Number of SNPs participated in the analysis; R^2^: The proportion of variance elucidated by instrumental variables (IVs) in the exposure variable in each trait pair; *P*a: *P*-value in IVW after multiple comparison BH correction; *P*(PRESSO): *P*-value after outlier-corrected in MR-PRESSO; Action = 3: Correct causal direction in MR Steiger test of directionality; SHBG: Sex hormone-binding globulin; TT: Total testosterone; BT: Bioactive testosterone; VTE: Venous thromboembolism; PE: Pulmonary embolism; DVT: Deep vein thrombosis

**Fig. 3:**
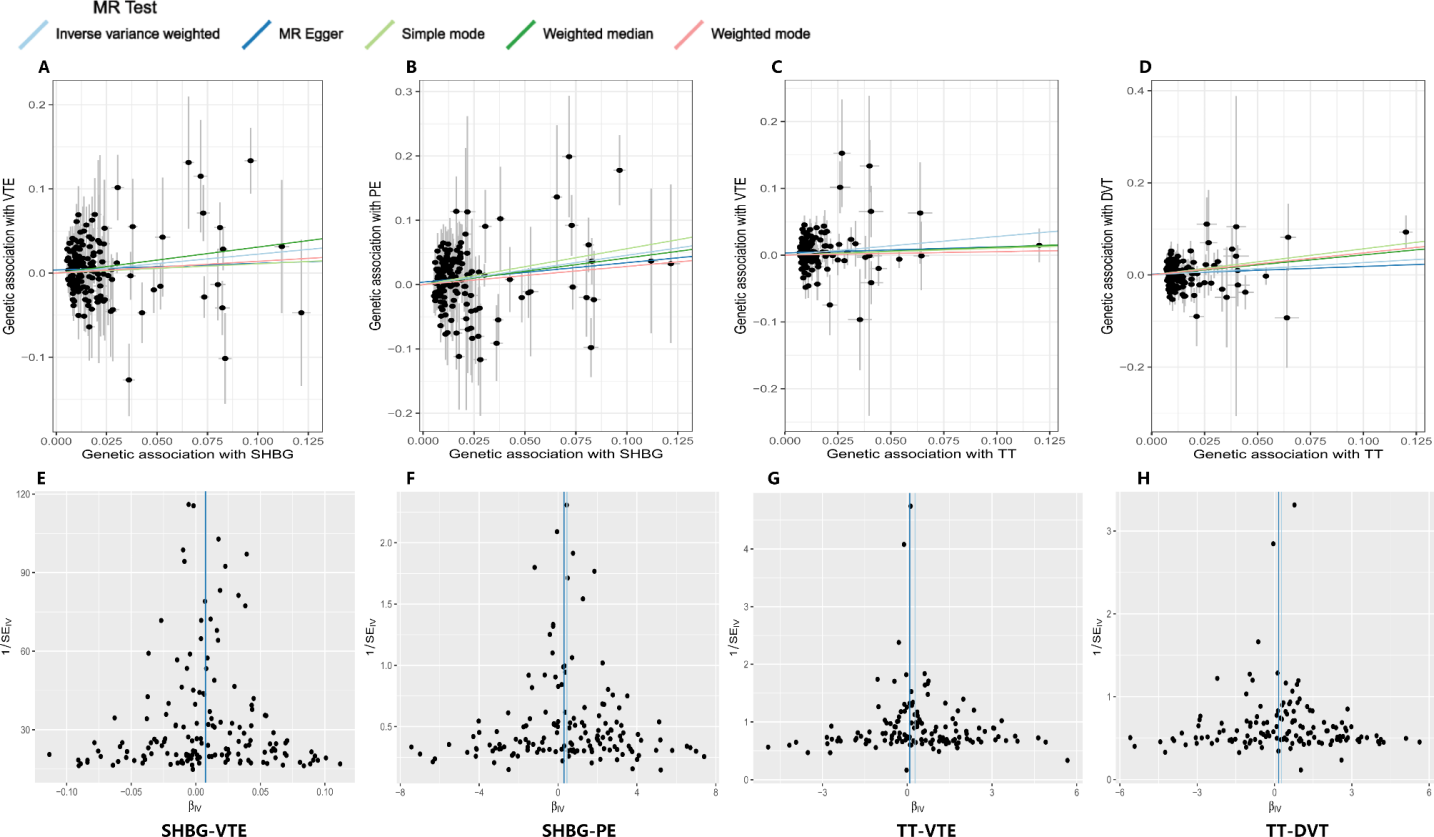
Scatter plots and funnel plots of the primary two-sample MR analyses (positive trait pairs). **(A-D)** In the scatter plots, the horizontal axis denotes the impact of instrumental variables (IVs) on the sex hormones, and the vertical axis represents the effect of IVs on VTE. Each black dots represents an individual SNP and the vertical and horizontal lines represent its corresponding 95% confidence interval (CI). The slope of the line represents the estimated causal effect of the various MR methods. **(E-H)** The horizontal axis of the funnel plot represents the estimated effect of each SNP on the exposure variable (β_IV_), while the vertical axis of 1/SE_IV_ reflects the precision or uncertainty of these estimates. MR: Mendelian randomisation; SHBG: Sex hormone-binding globulin; TT: Total testosterone; VTE: Venous thromboembolism; PE: Pulmonary embolism; DVT: Deep vein thrombosis

We conducted MR analysis to investigate the causal effects of SHBG, TT, and BT on VTE, PE, and DVT. As a result, nine trait pairs were analysed, four of which exhibited statistical differences before BH correction (*P*-value in both IVW and MR-PRESSO < 0.05). An increase in serum SHBG levels by one standard deviation (SD) caused 25% higher odds of VTE (OR: 1.25, 95%, CI: 1.01−1.55, *P*(IVW) = 0.0391, *P*(PRESSO) = 0.0409), and 58% higher odds for PE (OR: 1.58, 95%, CI: 1.20−2.08, *P*(IVW) = 0.0011, *P*(PRESSO) = 0.0014). An increase in serum total testosterone levels by one SD caused 32% higher odds for VTE (OR: 1.32, 95%, CI: 1.08−1.62, *P*(IVW) = 0.0068, *P*(PRESSO) = 0.0075), and 31% higher odds for DVT (OR, 1.31; 95%, CI: 1.01−1.69, *P*(IVW) = 0.0400, *P*(PRESSO), 0.0483). Two of the four pairs (TT-VTE and SHBG-PE) exhibited significant differences after BH-correct. The MR Steiger test of directionality showed ‘TRUE’ for all nine trait pairs. All the results are shown in Fig. 2 and Additional File 1 (Supplementary Table S2). Forest plots are shown in Fig. 2 and Additional File 2 (Supplementary Fig. 1). Scatter and funnel plots of the four positive trait pairs are shown in Fig. 3, and those of the negative trait pairs are shown in Additional File 2 (Supplementary Fig. 2 and Supplementary Fig. 3, respectively).

### MR sensitivity analysis

**Fig. 4:**
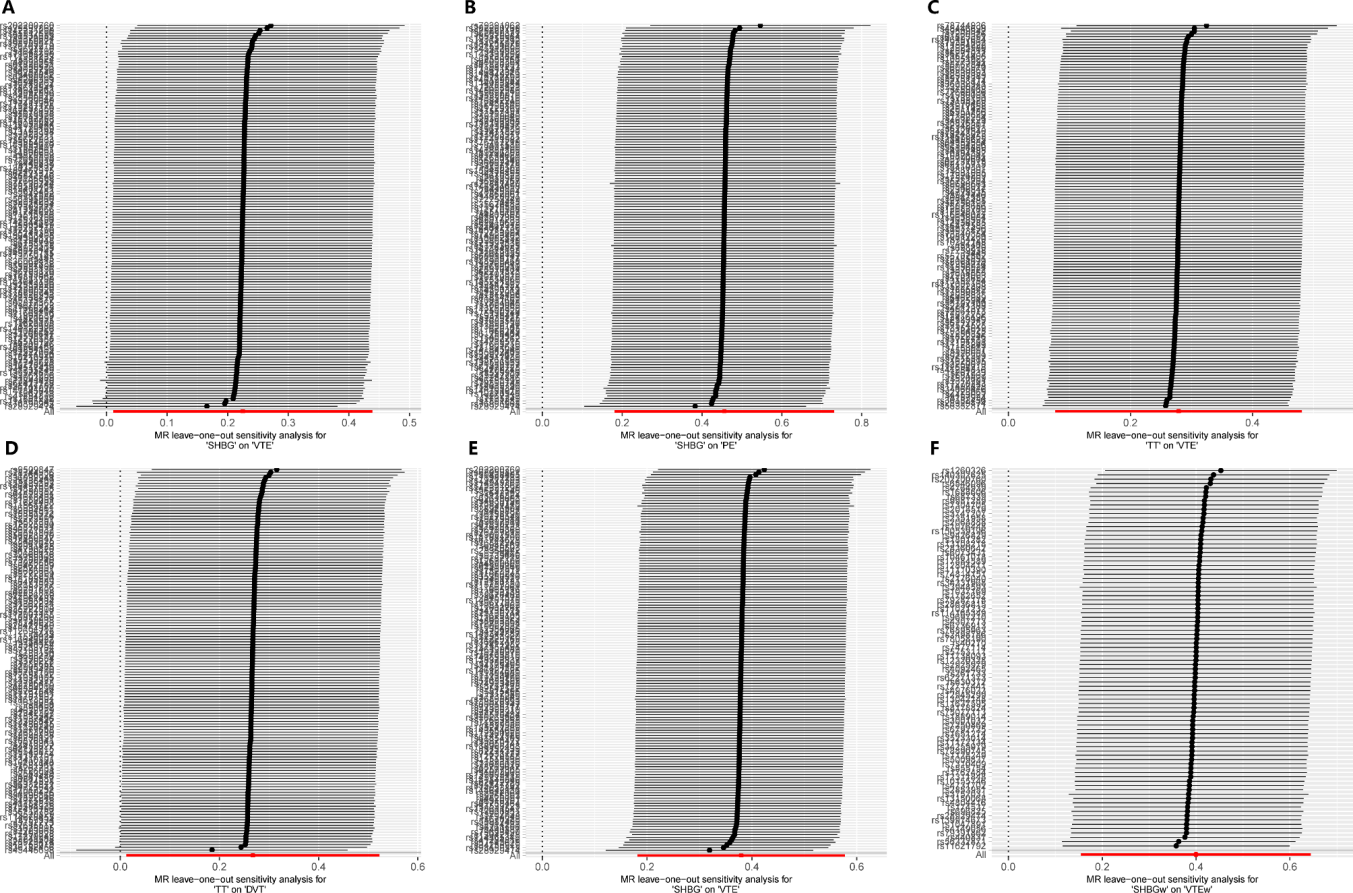
Leave-one-out plots of primary **(A-D)** and replicated **(E, F)** two-sample MR analyses (positive trait pairs). Each SNP is sequentially excluded to evaluate its impact on the causal estimate. MR: Mendelian randomisation; SHBG: Sex hormone-binding globulin; TT: Total testosterone; VTE: Venous thromboembolism; PE: Pulmonary embolism; DVT: Deep vein thrombosis; SHBGw: Sex hormone-binding globulin in women; VTEw: Venous thromboembolism in women

**Fig. 5:**
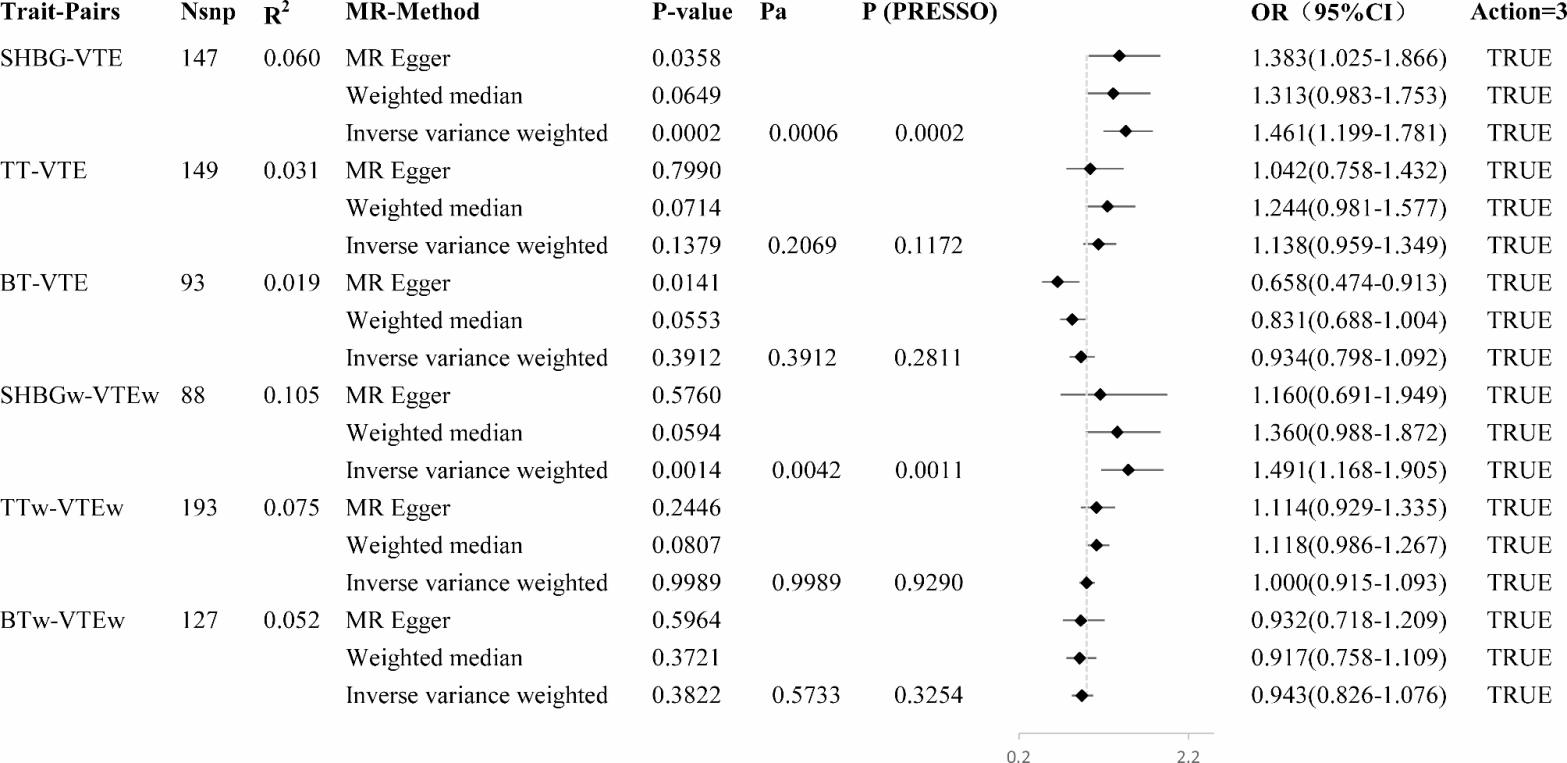
Causal relationships between sex hormones and VTE in replicated two-sample MR analyses. The effect of a one-standard deviation (SD) increase in sex hormone levels on the odds ratio (OR) of VTE is represented by the OR and its corresponding 95% confidence interval (CI). MR: Mendelian randomisation; Nsnp: Number of SNPs participated in the analysis; R^2^: The proportion of variance elucidated by instrumental variables (IVs) in the exposure variable in each trait pair; *P*a: *P*-value in IVW after multiple comparison BH correction; *P*(PRESSO): *P*-value after outlier-corrected in MR-PRESSO; Action = 3: Correct causal direction in MR Steiger test of directionality; SHBG: Sex hormone-binding globulin; TT: Total testosterone; BT: Bioactive testosterone; SHBGw: Sex hormone-binding globulin in women; TTw: Total testosterone in women; BTw: Bioactive testosterone in women; VTE: Venous thromboembolism; VTEw: Venous thromboembolism in women

**Fig. 6:**
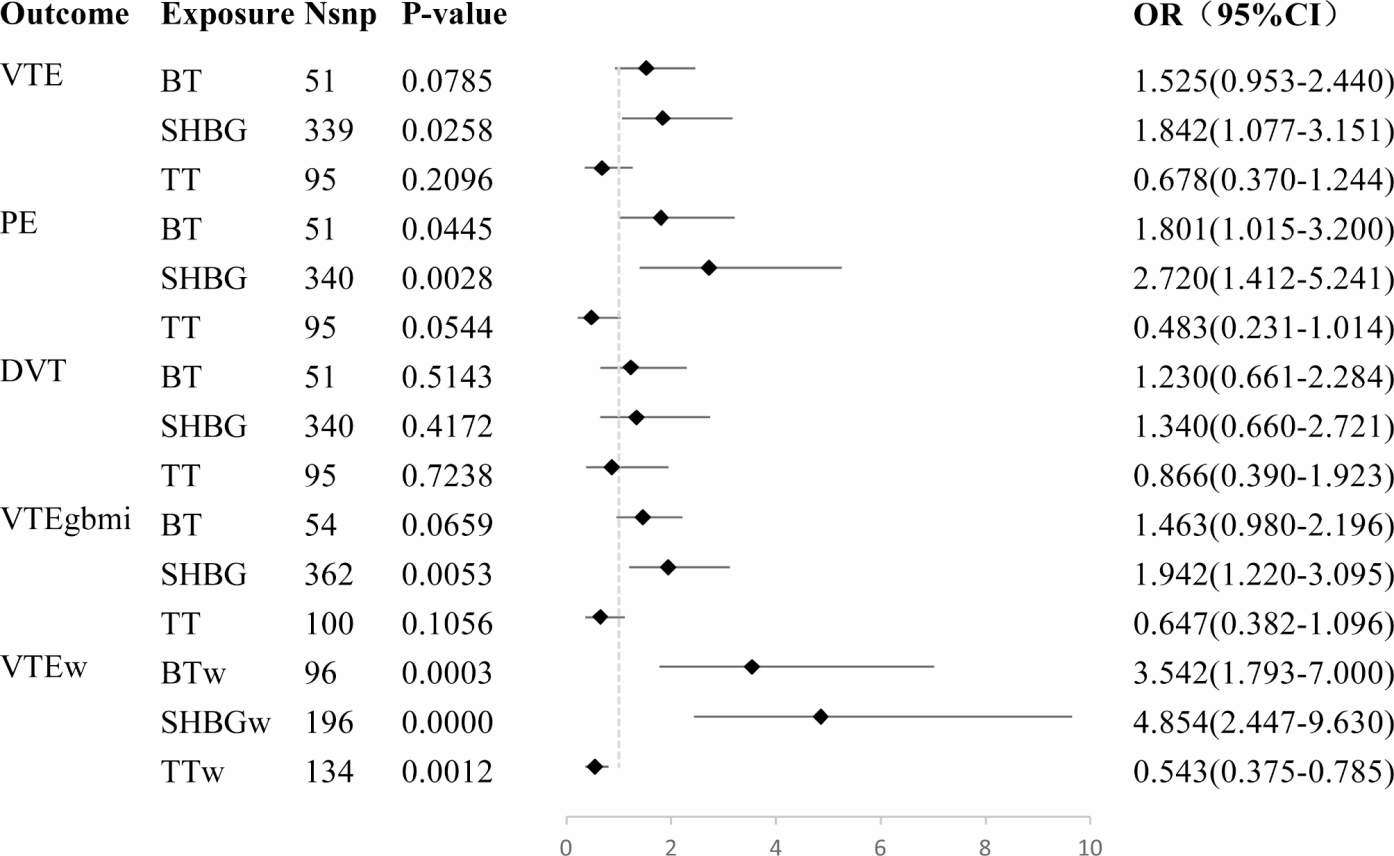
Causal relationships between sex hormones and VTE in multivariable Mendelian randomisation (MVMR). MR: Mendelian randomisation; Nsnp: Number of SNPs participated in analyses; SHBG: Sex hormone-binding globulin; TT: Total testosterone; BT: Bioactive testosterone; SHBGw: Sex hormone-binding globulin in women; TTw: Total testosterone in women; BTw: Bioactive testosterone in women; VTE: Venous thromboembolism; VTEgbmi: Venous thromboembolism from GBMI database in replicative MR analysis; VTEw: Venous thromboembolism in women; PE: Pulmonary embolism; DVT: Deep vein thrombosis

When assessing horizontal pleiotropy, the different MR methods (IVW, MR-Egger, and median MR) showed directionally consistent results, as shown in Fig. 2 and Additional File 1 (Supplementary Table S2), and all MR-Egger regression intercepts remained at zero without deviation, as shown in Table 1 and Additional File 1 (Supplementary Table S2). Moreover, the IVs exhibited no signs of horizontal pleiotropy, as indicated by intercept *P*-values exceeding 0.05, as shown in Table 1 and Additional File 1 (Supplementary Table S2). The leave-one-out analysis confirmed that no particular IVs drove any causal relationships, as shown in the leave-one-out plot (Fig. 4 and Additional File 2, Supplementary Fig. 4). In the LDSC results, three trait pairs showed significant genetic correlations (*P*-LDSC ≤ 0.05, Rg ≥ 0.1): SHBG-VTE, SHBG-PE, and SHBG-DVT. None of the nine trait pairs showed significant sample overlap in LDSC (*P* ≥ 0.05). As shown in Table 2. Replicated MR analyses were performed to verify the causal effect of SHBG, TT, and BT on VTE using data from both sexes and females from the GBMI Database. An increase in serum SHBG levels by one SD caused 46% higher odds for VTE (OR: 1.46, 95%, CI: 1.20−1.78, *P*(IVW) = 0.0002, *P*(PRESSO) = 0.0002) in both sexes and 49% higher odds for VTE (OR: 1.49, 95%, CI: 1.17−1.91, *P*(IVW) = 0.0014, *P*(PRESSO) = 0.0011) in females specifically, as shown in Fig. 5 and Additional File 1 (Supplementary Table S2), which further confirmed the results of the two-sample MR. MVMR analysed the causality between sex hormones as potentially relevant exposure variables; VTE, PE, and DVT from the Finnish database; and VTE from the GBMI database as outcomes. With outcome data from the Finnish database, the results showed a significant causal effect of SHBG on VTE (*P* = 0.0258) and PE (*P* = 0.0028) but not on DVT, which is consistent with the primary two-sample MR analyses, with the difference being that BT also showed a significant causal effect on PE (*P* = 0.0445). Using outcome data from the GBMI database, the results showed a significant causal effect of SHBG on VTE in both sexes (*P* = 0.0053) and females (*P* = 0.0000), which is also consistent with the replicative two-sample MR analyses. Additionally, female data showed a significant causal effect of BT (*P* = 0.0003) and TT (*P* = 0.0012) on VTE, as shown in Fig. 6 and Additional File 1 (Supplementary Table S3). Cochran’s Q test was performed to assess heterogeneity, and the *P*-values were below 0.05 (Table 1 and Additional File 1, Supplementary Table S2), indicating heterogeneity among the IVs and supporting the adoption of a random-effects model in the IVW analyses for these instances.

**Table 1:** Pleiotropy and Heterogeneity Tests for the Primary Two-sample Mendelian Randomisation (MR) Analyses

		Pleiotropy test			Heterogeneity test	
Trait-pairs	Nsnp	Int	SE	*P*-value	Q-*p*val	Q
SHBG-VTE	148	0.004	0.003	0.229	0.000	219.144
TT-VTE	138	0.003	0.003	0.221	0.000	231.745
BT-VTE	86	0.003	0.004	0.532	0.000	135.675
SHBG-PE	148	0.004	0.004	0.378	0.033	180.041
TT-PE	140	0.006	0.003	0.093	0.001	196.25
BT-PE	89	0.006	0.006	0.267	0.004	127.633
SHBG-DVT	147	0.007	0.004	0.080	0.019	183.602
TT-DVT	140	0.002	0.003	0.608	0.006	184.754
BT-DVT	91	0.002	0.006	0.663	0.000	147.133

Nsnp: Number of SNPs participated in the analysis; Int: Egger_intercept; SE: Standard error; Q: Heterogeneity statistic Q; SHBG: Sex hormone-binding globulin; TT: Total testosterone; BT: Bioactive testosterone; VTE: Venous thromboembolism; PE: Pulmonary embolism; VT: Deep vein thrombosis.

**Table 2:** Genetic correlation and genetic overlap estimated by Linkage Disequilibrium Score regression (LDSC)

	Genetic correlation			Genetic overlap			
Trait-pairs	Rg	Se	*P*-value	Beta	SE	*P*-value	
SHBG-VTE	0.1067	0.0362	0.0032	0.0004	0.0080	0.9601	
TT-VTE	0.0668	0.0377	0.0762	-0.0075	0.0055	0.1727	
BT-VTE	0.0454	0.0431	0.2918	0.0019	0.0060	0.7515	
SHBG-PE	0.1189	0.0487	0.0146	0.0004	0.0080	0.9601	
TT-PE	0.0997	0.0479	0.0375	-0.0053	0.0056	0.3439	
BT-PE	0.0537	0.0561	0.3384	0.0011	0.0053	0.8356	
SHBG-DVT	0.1334	0.0417	0.0014	-0.0018	0.0077	0.8152	
TT-DVT	0.0627	0.0426	0.1412	-0.0091	0.0059	0.1230	
BT-DVT	0.0367	0.0507	0.4691	-0.0026	0.0058	0.6540	
SHBG-VTE	0.1067	0.0362	0.0032	0.0004	0.0080	0.9601	

*P*-value ≤ 0.05 indicates that the assumptions of genetic association or genetic overlap hold true. Rg: Genetic correlation; SHBG: Sex hormone-binding globulin; TT: Total testosterone; BT: Bioactive testosterone; VTE: Venous thromboembolism; PE: Pulmonary embolism; DVT: Deep vein thrombosis.

## Discussion

In this study, we explored the genetic associations and causality of SHBG, TT, and BT with VTE, PE, and DVT using a combination of two-sample MR, MRPRESSO, and LDSC. In the primary MR analyses, we found that elevated serum SHBG levels increased the risk of VTE and PE, whereas elevated serum TT levels increased the risk of VTE and DVT. Furthermore, the LDSC and MR sensitivity analyses supported the causal effect of SHBG on VTE. However, the causal effects of testosterone levels have not been verified.

### Sex hormone-binding globulin (SHBG) and venous thromboembolism (VTE)

Previous studies have shown that the use of exogenous oestrogen, androgens, and endogenous oestrogen increases the risk of VTE. However, the association between endogenous androgens such as SHBG and VTE remains controversial.

Numerous investigations have substantiated the independent prognostic value of SHBG in assessing VTE risk subsequent to the use of hormonal contraceptives [19–21]. A recent case-control study involving women aged ≤ 45 years revealed that the occurrence risk of VTE exhibits a dose-response association with SHBG levels, indicating an OR of up to 2.0 for individuals in the highest quartile of SHBG levels compared to those in the lowest quartile, suggesting the pathophysiological effects of SHBG VTE in young women not using contraceptives [22]. In this study, primary IVW before BH correction and MR-PRESSO suggested a causal effect of SHBG on VTE. The replicated MR analyses in the largest VTE database currently knownand MVMR also supported this result, combined with the results of LDSC, which supports the genetic correlation between SHBG and VTE. We suggest that SHBG is causally associated with VTE, consistent with the conclusions of previous studies. Simultaneously, owing to the differences in sex hormones and SHBG between genders, we used the largest GWAS data available on female VTE to confirm the causal relationship between SHBG and VTE in females. Unfortunately, there are currently no available male VTE data that do not significantly overlap with our exposure data (VTE data for both males and females can be obtained in UKBB, but our previous LDSC analysis suggests a high overlap with exposure data). In addition, it must be emphasized that in the original GWAS study of the exposure data, the authors analysed and pointed out that the genetic structure of SHBG levels is highly consistent between males and females [16]; therefore, the results of the MR analysis using population-wide SHBG genetic IVs are credible, we concluded that a causal relationship exists between SHBG and VTE. Among the subtypes of VTE, SHBG was causally associated with PE in the primary MR and MVMR analyses, which is consistent with the genetic correlation of LDSC. However, we recognize that DVT and PE are considered two clinical presentations of VTE, with approximately 50–70% of PE patients concurrently experiencing DVT and 50% of DVT patients having an associated asymptomatic PE noted on lung scans [35]. The conclusion that SHBG is causally associated with PE but not DVT requires further validation. The transition from DVT to PE involves intricate physiological regulation, and several risk factors which predict the occurrence of PE in DVT patients, such as hypertension, diabetes, long lying state, glucocorticoid therapy, and D-dimer levels, have been reported [36, 37]. Therefore, our findings may allow us to speculate that SHBG could potentially be associated with a higher risk of PE development in DVT patients within the context of VTE formation. However, clinical research on the high-risk factors and underlying mechanisms for the occurrence of PE in DVT patients are limited. Our speculation in this regard necessitates further validation through subsequent research endeavours.

However, the mechanisms underlying the regulation of VTE by SHBG remain unclear. Among women using hormonal contraceptives, investigators have considered SHBG a marker of the ‘oestrogenicity’ of contraceptives, and oestrogen is an established risk factor for VTE [19]. Some researchers have also hypothesized that hepatically metabolized hormonal contraceptives may influence the production of SHBG and coagulation factors, ultimately contributing to VTE [20]. In addition, SHBG can be regarded as a potent amplifier of steroid activity, and it is the main transporter of hydrophobic androgens in hydrophilic blood, but perhaps less so for oestrogens, which can also undergo glucuronidation and sulfation to facilitate their transportation to target organs [38]. Therefore, their effects may also be via testosterone [39].Furthermore, the interaction between SHBG and megalin might act as an additional autocrine-controlled mechanism, actively transporting steroids into cells and potentially modulating hormone signalling pathways [38]. It is also believed that SHBG may be implicated in the modulation of chronic inflammation, cellular proliferation, and lipid metabolism, and these processes can potentially impact the development of VTE independent of sex hormones [17, 40]. Regardless of the underlying mechanism, SHBG may be a potential clinical biomarker of VTE. Follow-up studies should focus on exploring the pathophysiological mechanisms of SHBG in VTE to look forward to identifying novel therapeutic targets.

### Testosterone and venous thromboembolism (VTE)

Multiple observational studies have indicated a potential elevation in short-term VTE risk among men receiving testosterone therapy [6, 7, 41]. A recent case-crossover study demonstrated that testosterone therapy was associated with an elevated risk of VTE within 12 months of follow-up in men with and without hypogonadism, with an OR of 2.32 (95% CI, 1.97−2.74) and 2.02 (95% CI, 1.47−2.77), respectively [42]. The mechanism underlying this effect might be attributed to the elevation of Hct levels in men following testosterone treatment, which is significantly associated with VTE [7, 43]. The current observational findings do not support an association between endogenous testosterone levels and the risk of VTE in men or women [10, 11, 22, 44]. However, a recent MR study observed a positive association between endogenous testosterone, genetically predicted by mutations in the JMJD1C gene region, and thromboembolism (VTE, arterial embolism, and thrombosis) (OR: 2.09, 95% CI: 1.27−3.46) in men, whereas no such association was found in women, and no association was observed between endogenous testosterone, genetically predicted by mutations in the SHBG gene region, and thromboembolism [45]. However, similar to this study, the correlation between testosterone and VTE failed to obtain consistent results in validation studies. Coupled with the small sample size (3225 men), we believe that their conclusions need to be further verified. In this study, the results of both IVW and MR-PRESSO suggested a causal relationship between TT but not BT and VTE. However, the replicated MR analyses in both sexes and females did not support this result, nor did the MVMR data in either sex or LDSC. Additionally, in the original GWAS of exposure data, the authors highlighted that the genetic contribution to the variability in circulating testosterone levels varies considerably between males and females, with several variants having genome-wide significance for testosterone in opposite directions, thus emphasising that sex-disaggregated data are best used in genetic association analyses of testosterone [16]. Additionally, in our MVMR analyses of female data, TT and BT had a significant causal effect on VTE. Therefore, we cannot completely exclude the correlation between testosterone levels and VTE when conducting sex-specific analyses. In conclusion, although this study had a larger sample size than previous MR studies, given the negative results of the LDSC and replicated analyses and the lack of better available sex-disaggregated data, we considered whether there was a causal association between testosterone and VTE needs the support of further large-sample sex-disaggregated MR analysis results.

### Limitations and prospects

We conducted the first MR study to confirm the genetically predicted causal relationship between SHBG and VTE; however, this study had a few limitations. First, because the three assumptions of MR studies cannot be precisely satisfied, corresponding bias cannot be eliminated. In terms of instrument relevance, we adopted a stricter *P*-value (*P* < 5 × 10^− 8^), calculated the F value of each SNP, and deleted SNPs with F ≤ 10. The R^2^ of the IVs used in all MR analyses ranged from 1.9 to 10.5%, thereby reducing the bias introduced by weak IVs. Horizontal pleiotropy was the main reason for the bias in instrument validity. We used MR-PRESSO to exclude outliers that may have pleiotropic effects and then performed a subsequent MR analysis. Although IVW was utilized as the principal methodology to ascertain causal associations, we ensured the consistency of direction using various MR analysis methods. In addition, the leave-one-out analyses showed no significant horizontal pleiotropy, and the MVMR analyses showed robust results, consistent with the main MR analysis of the overall results, which were mainly used to rule out bias owing to the correlation between sex hormones. Additionally, we performed replicated MR analyses using an independent outcome database and LDSC to confirm the genetic correlation and reliability of primary MR analyses. Reverse causality was ruled out using MR Steiger. Second, heterogeneity tests revealed some heterogeneity in this study, which may reflect the different responses of different populations or environments to research effects. In response, a random-effects model was applied for IVW analyses. Third, this study was conducted in a European population; therefore, generalising the results to other races requires further verification. Fourth, sex hormones differ between sexes, and the genetic IVs of testosterone also show large differences. However, owing to the lack of sex-specific outcome data that did not overlap with our exposure data, our sex-disaggregated data were only for women. Therefore, the correlation between testosterone levels and VTE was used as the reference in this study. However, because the genetic IVs of SHBG described by the original author were not significantly different between the sexes, the results of this study on SHBG remain credible. Further, large-sample sex-disaggregated MR analyses of sex hormones, especially testosterone, in VTE are necessary to determine their causal relationship. Therefore, SHBG may be a potential clinical biomarker for VTE, and follow-up studies should focus on exploring the pathophysiological mechanisms of SHBG in VTE to look forward to developing novel therapeutic targets.

## Conclusions

Our MR study suggests that elevated serum SHBG levels predicted by genetics may increase the risk of VTE; however, the causal relationship between testosterone and VTE requires further research. SHBG may play a pathological role in VTE and can be used as a predictor of lifetime risk and a novel therapeutic target for VTE. However, further studies of this mechanism are required.

### Electronic supplementary material

Below is the link to the electronic supplementary material.

Supplementary Material 1

Supplementary Material 2

## Data Availability

The exposure data supporting the conclusions of this article are openly accessible and available in the UK Biobank (https://www.nealelab.is/uk-biobank). The outcome data in primary MR analyses are openly accessible and available in R8 release of the FinnGen GWAS results (https://r8.finngen.fi). The outcome data in replicated MR analyses are openly accessible and available in GBMI database(https://www.globalbiobankmeta.org/).

## Abbreviations

BH: Benjamini-Hochberg
BMI: Body mass index
BT: Bioactive testosterone
CI: Confidence interval
DVT: Deep vein thrombosis
GBMI: Global Biobank Meta-analysis Initiative
GWAS: Genome-wide association study
ICD: International Classification of Diseases
IVs: Instrumental variables
IVW: Inverse variance weighting
LDSC: Linkage disequilibrium score regression
MR: Mendelian randomisation
MR-PRESSO: MR-pleiotropy residual sum and outlier
MVMR: Multivariable Mendelian randomization
OR: Odds ratio
R^2^: Percentage of the variation explained by the IVs
PE: Pulmonary embolism
SD: Standard deviation
SHBG: Sex hormone-binding globulin
SNP: Single nucleotide polymorphisms
TT: Total testosterone
VTE: Venous thromboembolism
