# Supplementary material for: Causal inference of sex hormone-binding globulin on venous thromboembolism: evidence from Mendelian randomisation
Source: Thromb J. 2023 Oct 25;21:109. doi: 10.1186/s12959-023-00553-9 (PMC10599068; doi:10.1186/s12959-023-00553-9)
Supplement: Supplementary file 2 — Supplementary Material 2 [file 12959_2023_553_MOESM2_ESM.docx]

**Supplementary Fig.1** Forest plots of the primary two-sample MR analyses. SHBG: Sex hormone-binding globulin; TT: Total testosterone; BT: Bioactive testosterone; VTE: Venous thromboembolism; PE: Pulmonary embolism; DVT: Deep vein thrombosis.


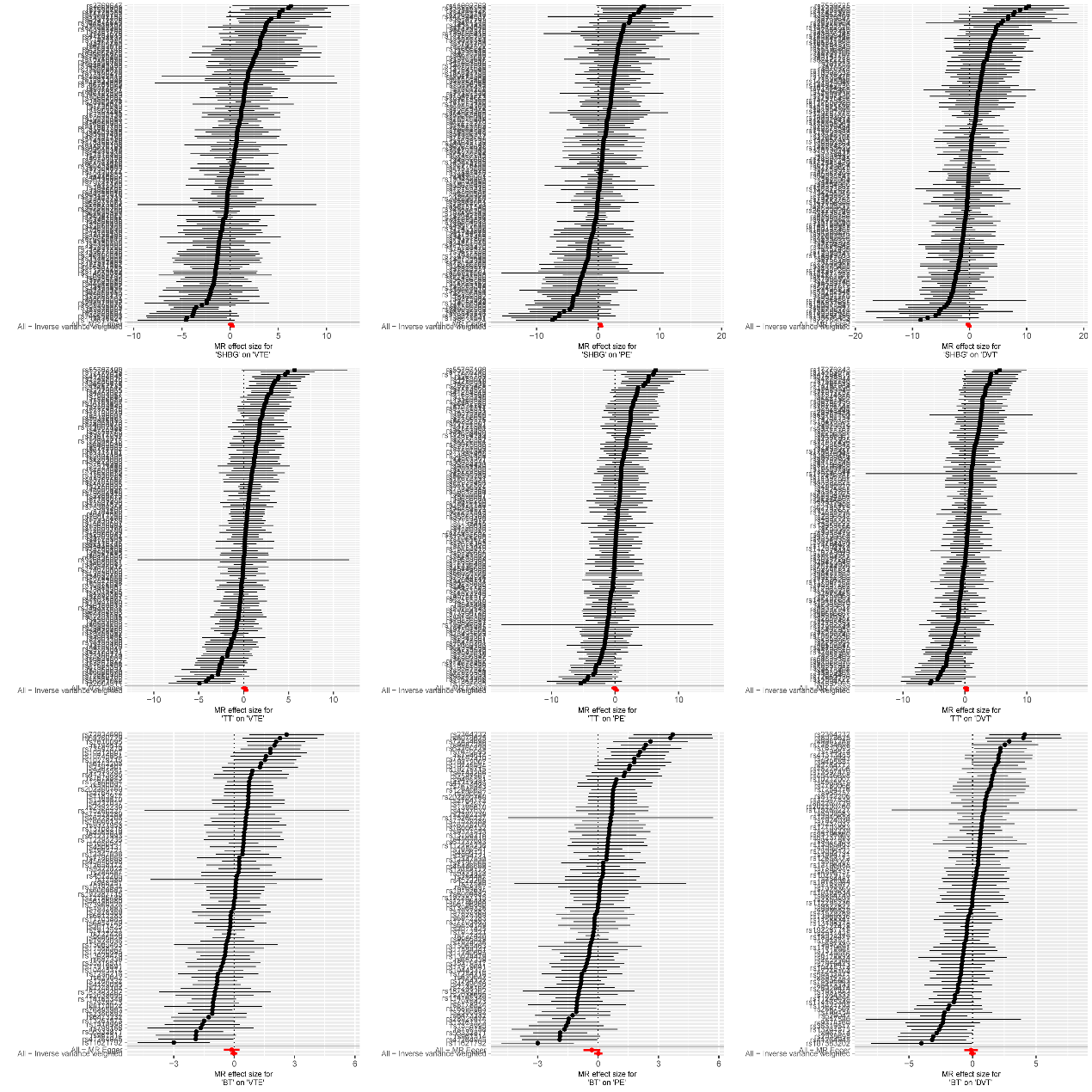


**Supplementary Fig.2** Scatter plots of primary(A-E) and replicative(F-I) two-sample MR analyses (negative trait pairs). The horizontal axis denotes the impact of instrumental variables (IVs) on the sex hormones, and the vertical axis represents the effect of IVs on VTE. Each black dots represents an individual SNP and the vertical and horizontal lines represent its corresponding 95% confidence interval (CI). The slope of the line represents the estimated causal effect of the various MR methods. SHBG: Sex hormone-binding globulin; TT: Total testosterone; BT: Bioactive testosterone; SHBGw: Sex hormone-binding globulin in women; TTw: Total testosterone in women; BTw: Bioactive testosterone in women; VTE: Venous thromboembolism; VTEw: Venous thromboembolism in women; PE: Pulmonary embolism; DVT: Deep vein thrombosis.


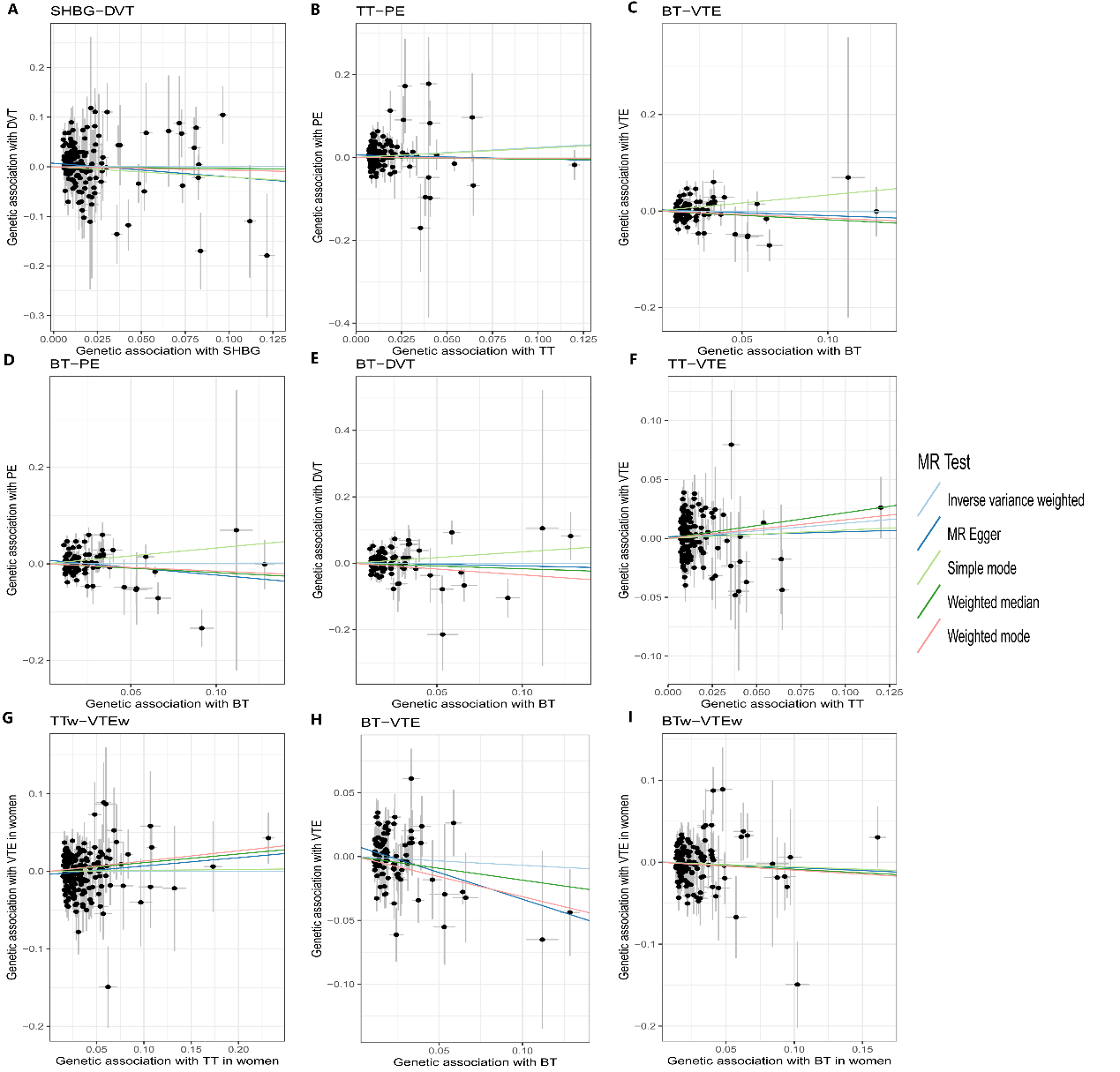


**Supplementary Fig.3** Funnel plots of primary(A-E) and replicative(F-I) two-sample MR analyses (negative trait pairs). The horizontal axis of the funnel plot represents the estimated effect of each SNP on the exposure variable (β_IV_), while the vertical axis of 1/SE_IV_ reflects the precision or uncertainty of these estimates. SHBG: Sex hormone-binding globulin; TT: Total testosterone; BT: Bioactive testosterone; SHBGw: Sex hormone-binding globulin in women; TTw: Total testosterone in women; BTw: Bioactive testosterone in women; VTE: Venous thromboembolism; VTEw: Venous thromboembolism in women; PE: Pulmonary embolism; DVT: Deep vein thrombosis.


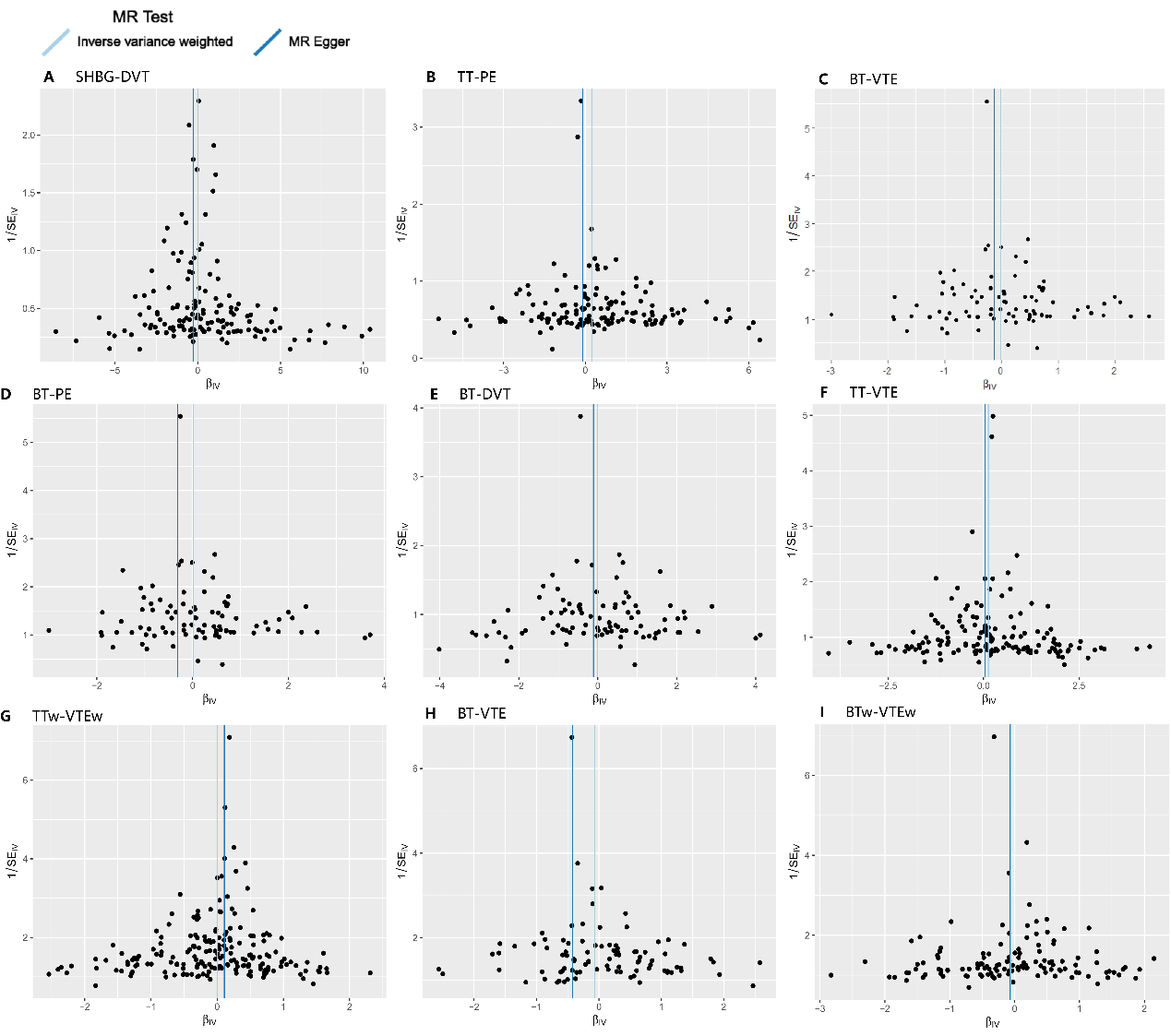


**Supplementary Fig.4** Leave-one-out plots of primary (A-E) and replicative (F-I) two-sample MR analyses (negative trait pairs). Each SNP is sequentially excluded to evaluate its impact on the causal estimate. SHBG: Sex hormone-binding globulin; TT: Total testosterone; BT: Bioactive testosterone; SHBGw: Sex hormone-binding globulin in women; TTw: Total testosterone in women; BTw: Bioactive testosterone in women; VTE: Venous thromboembolism; VTEw: Venous thromboembolism in women; PE: Pulmonary embolism; DVT: Deep vein thrombosis.


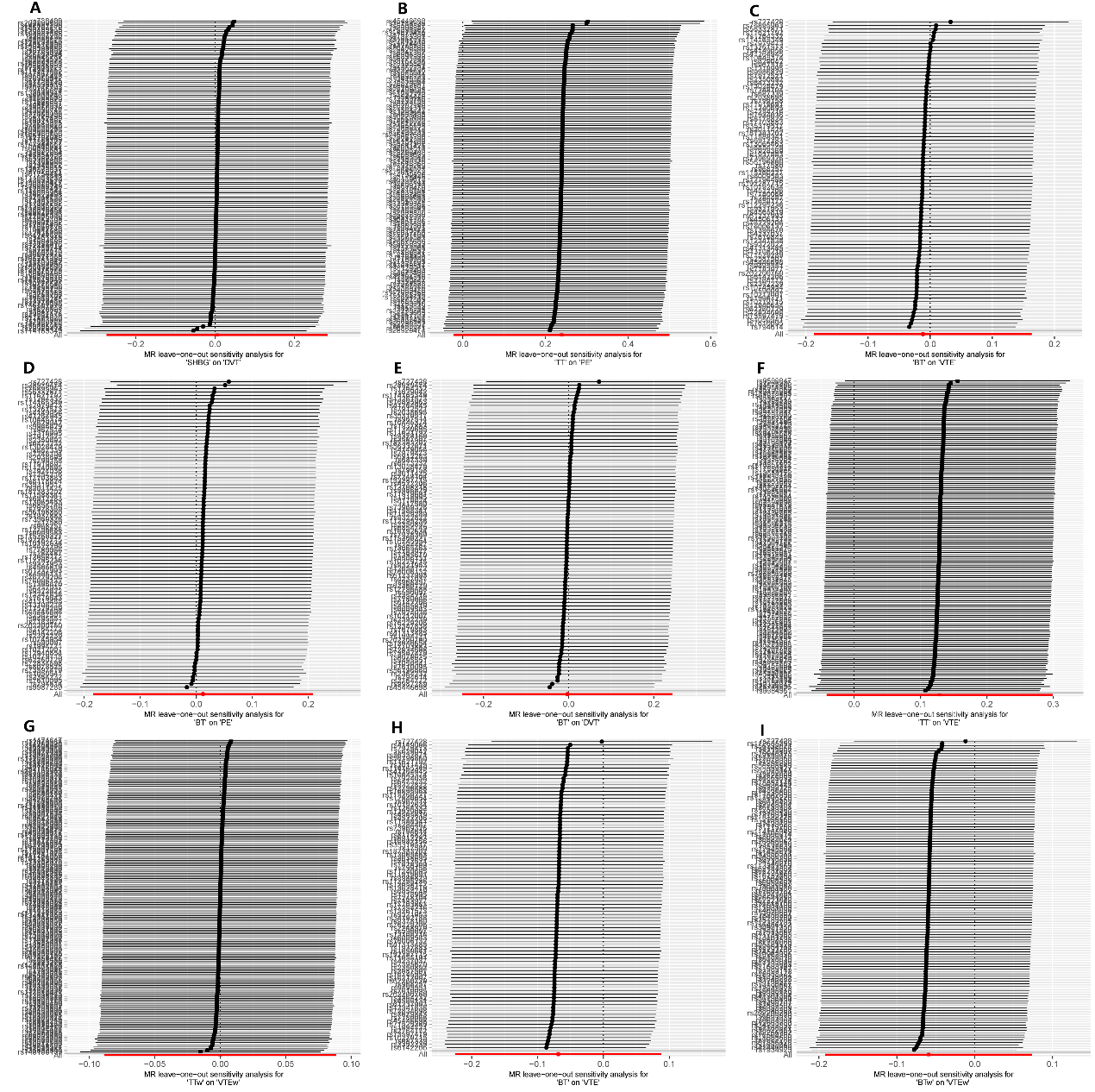


**Supplementary Fig.5** Forest plots of the replicative two-sample MR analyses. SHBG: Sex hormone-binding globulin; TT: Total testosterone; BT: Bioactive testosterone; SHBGw: Sex hormone-binding globulin in women; TTw: Total testosterone in women; BTw: Bioactive testosterone in women; VTE: Venous thromboembolism; VTEw: Venous thromboembolism in women.


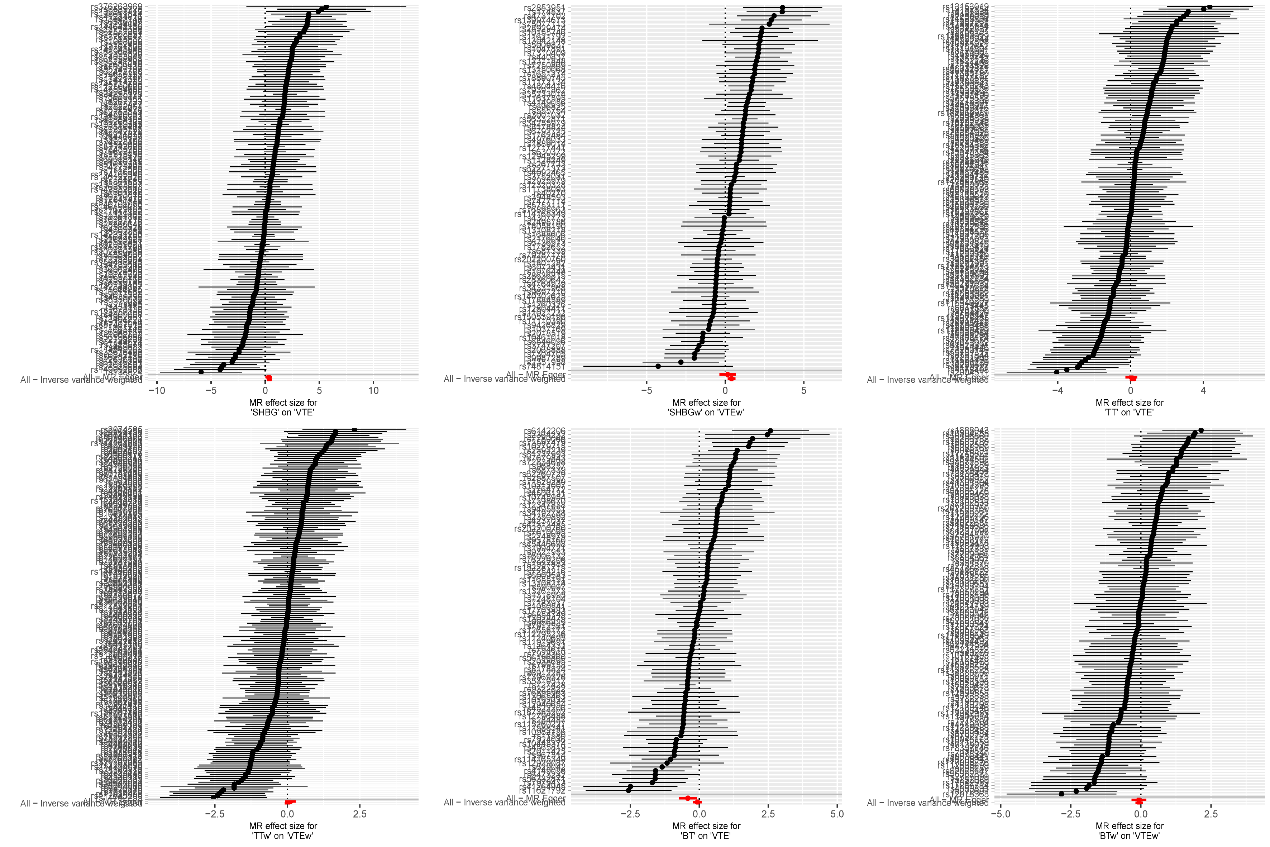


**Additional File 1 Supplementary Table S1:** Genetic instrumental variables (IVs) used in primary and replicative two-sample MR analyses. SHBG: Sex hormone-binding globulin; TT: Total testosterone; BT: Bioactive testosterone; SHBGw: Sex hormone-binding globulin in women; TTw: Total testosterone in women; BTw: Bioactive testosterone in women; VTE: Venous thromboembolism; VTEgbmi: Venous thromboembolism from GBMI in replicative analysis; VTEw: Venous thromboembolism in women; PE: Pulmonary embolism; DVT: Deep vein thrombosis.

**Additional File 1** **Supplementary Table S2:** Primary and replicative two-sample MR and sensitivity analyses. SHBG: Sex hormone-binding globulin; TT: Total testosterone; BT: Bioactive testosterone; SHBGw: Sex hormone-binding globulin in women; TTw: Total testosterone in women; BTw: Bioactive testosterone in women; VTE: Venous thromboembolism; VTEgbmi: Venous thromboembolism from GBMI in replicative analyses; VTEw: Venous thromboembolism in women; PE: Pulmonary embolism; DVT: Deep vein thrombosis.

**Additional File 1** **Supplementary Table S3:** Multivariable mendelian randomization (MVMR). SHBG: Sex hormone-binding globulin; TT: Total testosterone; BT: Bioactive testosterone; SHBGw: Sex hormone-binding globulin in women; TTw: Total testosterone in women; BTw: Bioactive testosterone in women; VTE: Venous thromboembolism; VTEgbmi: Venous thromboembolism from GBMI in replicative analysis; VTEw: Venous thromboembolism in women; PE: Pulmonary embolism; DVT: Deep vein thrombosis.
